# HbA1c mediates association between masticatory function and hypertension results from NHANES: An observational study

**DOI:** 10.1097/MD.0000000000046772

**Published:** 2026-01-02

**Authors:** Hao Guo, Hang Yang, Jie Li, Tian Lv

**Affiliations:** a Department of Cardiology, Zhuji Affiliated Hospital of WenZhou Medical University, Shaoxing, Zhejiang Province, China; b Department of Stomatology, Stomatology Hospital, School of Stomatology, Zhejiang University School of Medicine, Hangzhou, Zhejiang Province, China; c Department of Neurology, Wenzhou Medical University Lishui Hospital, Lishui People’s Hospital, Lishui, Zhejiang Province, China; d Department of Neurology, Zhuji Affiliated Hospital of WenZhou Medical University, Shaoxing, Zhejiang Province, China.

**Keywords:** functional tooth units, HbA1c, hypertension, masticatory function

## Abstract

Hypertension constitutes a widespread public health concern. While epidemiological evidence has linked tooth loss with hypertension, the precise association between masticatory function operationally defined through functional tooth units (FTUs) and hypertension remains insufficiently elucidated. The aim to investigate the relationship between masticatory function and hypertension. This study analyzed data from the National Health and Nutrition Examination Survey (NHANES) spanning 2005–2018. Hypertension status was ascertained through standardized questionnaires and triplicate blood pressure measurements. Masticatory function, defined as FTUs, was characterized by the presence of antagonistic natural or prosthetic tooth pairs in premolar and molar regions. To investigate the FTUs-hypertension association, we employed logistic regression models coupled with restricted cubic spline analyses. Subgroup stratification and mediation analysis were subsequently conducted to elucidate potential effect modifiers and biological mechanisms. Individuals with optimal masticatory function (10 ≤ FTUs ≤ 12) exhibited an 18% reduction in hypertension risk compared to those with compromised function (FTUs < 3), demonstrating an adjusted odds ratio of 0.82 (95% confidence interval: 0.72–0.92, *P* < .0001). Mediation analysis identified metabolic markers: glycated hemoglobin as a significant intermediary, accounting for 18.0% (95% confidence interval: 14.8–21.1) of the total effect. Subgroup analyses revealed significant effect modification by gender and age (*P*-interaction < .05). Restricted cubic spline modeling demonstrated a nonlinear association, with hypertension risk following an inverted U-shaped curve across FTU quintiles (*P*-nonlinearity < .001). Our findings suggest that higher FTUs may lower the risk of hypertension via the metabolic markers: glycated hemoglobin pathway.

## 1. Introduction

Hypertension persists as a leading modifiable cardiovascular risk factor for premature mortality globally, serving as an integral component of the World Health Organization’s (WHO) Global Action Plan targets for non-communicable disease prevention and control.^[1]^ Spanning the period 1990 to 2019, the global burden of hypertension manifested a fourfold escalation, with the worldwide prevalence surpassing the 1 billion threshold according to WHO surveillance data.^[2]^

Tooth loss represents the primary etiological factor underlying compromised masticatory function.^[3]^ Masticatory funtion demonstrates significant systemic associations with overall physiological homeostasis.^[4]^ Impaired masticatory function may induce dietary pattern modifications and disrupt nutritional equilibrium, manifesting as reduced consumption of fruits/vegetables, preferential selection of high-fat/low-fiber diets, and suboptimal intake of essential proteins and vitamins,^[5–7]^ these cumulative dietary deviations can precipitate malnutrition, thereby increasing predisposition to multisystem chronic disorders (such as hypertension or diabetes) and all-cause mortality.^[8–12]^

A growing body of research has established a robust positive association between tooth loss and hypertension onset.^[13–16]^ However, the number of missing teeth does not reliably reflect the extent of masticatory function impairment. Masticatory function becomes significantly compromised exclusively when paired antagonistic tooth loss occurs within the premolar and molar regions.^[17]^ Functional Tooth Units (FTUs), defined as paired antagonistic teeth within the premolar and molar regions,^[18]^ serve as critical biomarkers for evaluating masticatory function. These occlusal pairs constitute primary determinants of chewing efficiency, with their functional integrity directly influencing nutritional intake capacities.^[19,20]^ Our study addressed the current knowledge gap regarding the absence of a nationally representative sample to study the relationship between FTUs and hypertension. Studies indicate that mastication regulates blood glucose through multiple mechanisms, and individuals with greater chewing ability exhibit a lower prevalence of diabetes.^[21]^ Diabetes contributes to the development of hypertension by increasing peripheral vascular resistance through vascular remodeling and by elevating fluid volume associated with hyperinsulinemia and hyperglycemia.^[22]^ Epidemiological studies have demonstrated that patients with type 2 diabetes have a 2.0- to 2.5-fold higher incidence of hypertension compared with those without type 2 diabetes.^[23]^ Therefore, we incorporated metabolic markers: glycated hemoglobin (HbA1c) as a mediating factor to investigate the association between FTUs and hypertension.

The purpose of this study was to investigate the relationship between masticatory function and hypertension in the population using data from National Health and Nutrition Examination Survey in 2005–2018 (NHANES).

## 2. Materials and methods

### 2.1. Study design and population

NHANES data are provided by the US Centers for Disease Control and Prevention, National Center for Health Statistics website. The data were derived from the NHANES. Our analysis utilized 2005–2018 NHANES data comprising cross-sectional, nationally representative samples of non- institutionalized US adults. During standardized clinical examinations, certified technicians obtained anthropometric measurements (height and weight) and blood pressure readings; concurrent biospecimen collection included blood and urine samples for laboratory analyses. The study protocol received institutional review board approval, and written informed consent was obtained from all participants.

**Figure 1.**
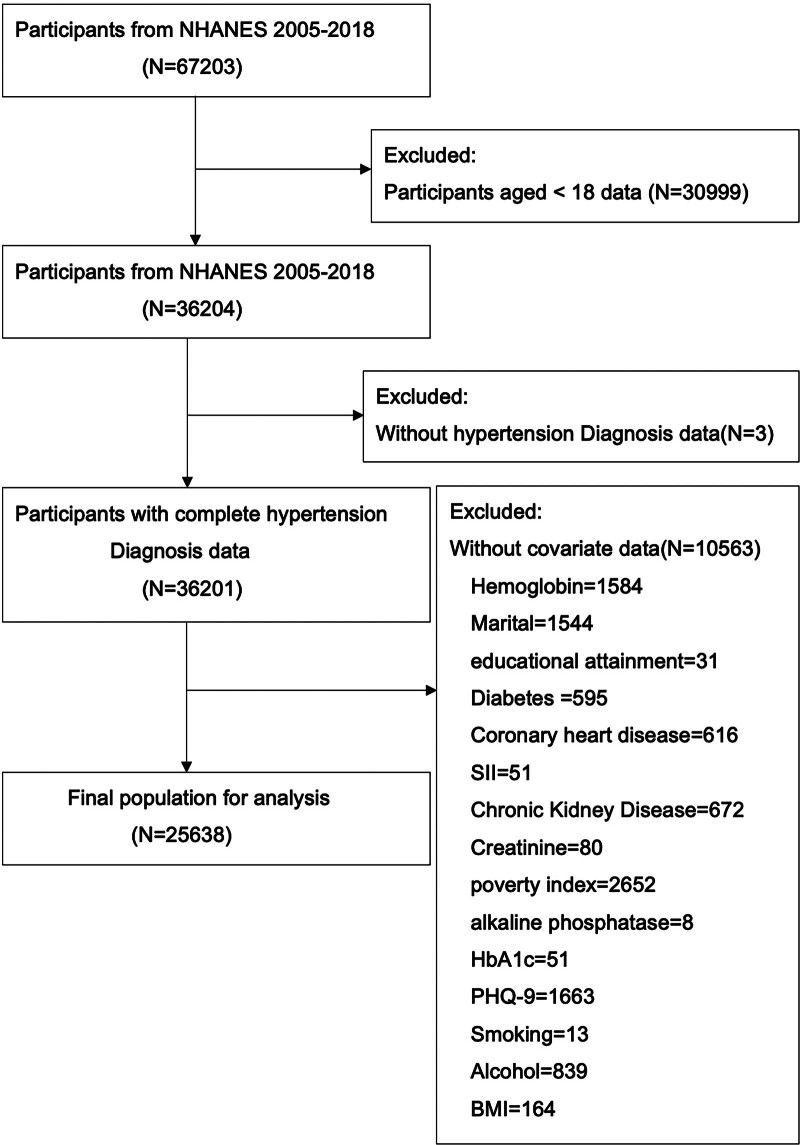
Flowchart of participant enrollment process. BMI = body mass index, HbA1c = metabolic markers: glycated hemoglobin, NHANES = National Health and Nutrition Examination Survey, PHQ-9 = 9-item Patient Health Questionnaire.

Participants were included if data were available for both FTU calculation, hypertension diagnosis and complete diagnosis data. Participants younger than 18 years, or those lacking hypertension diagnosis data or covariate data, were excluded from the analyses (Fig. 1).

#### 2.1.1. Assessment of hypertension

Hypertension was defined as systolic blood pressure ≥ 140 mm Hg, diastolic blood pressure ≥ 90 mm Hg, self-reported hypertension, or current use of antihypertensive medication.

#### 2.1.2. Assessment of FTUs

The number of FTUs was defined as pairs of opposing natural and artificial teeth (implant-supported, fixed and removable prostheses), third molars excluded from the calculation. The scoring system assigns: 1 FTU for 2 opposing premolars and 2 FTUs for 2 opposing molars.^[24]^ The calculation of FTUs in this study was constrained by methodological limitations inherent to NHANES datasets, specifically the exclusion of tooth mobility assessments. Consequently, FTU determination relied exclusively on static dentition status.

#### 2.1.3. Covariates

This study incorporated covariates encompassing 3 principal domains: demographic parameters, biochemical indices, and comorbid conditions, all obtained via structured self-administered questionnaires. Demographic variables were systematically stratified into: biological factors: age and gender (male/female); socioeconomic determinants: educational attainment (categorized as below high school, high school graduate, or college-level education), marital status (married/unmarried), and poverty index; behavioral characteristics: smoking status (never/former/current) and alcohol status (never/heavy/mild/moderate/heavy). Race/ethnicity was classified as White, Black, or other ethnicities. FTUs were analyzed as a discrete dental health metric. Depressive symptomatology was evaluated using the 9-item Patient Health Questionnaire (PHQ-9), with clinically significant depression defined as a composite score ≥ 10.^[25]^ Body mass index (BMI) was quantified as the quotient of body weight (kg) to squared height (m²). Biochemical parameters comprised: HbA1c and creatinine; hepatic function indicators: alkaline phosphatase and albumin; and hematological components: hemoglobin level, platelet count, and differential leukocyte counts (lymphocytes, monocytes, neutrophils). The systemic immune-inflammation index (SII) was calculated using the formula: SII = (platelet count × neutrophil count) ÷ lymphocyte count, as validated in prior immunological studies.^[26]^

Coronary heart disease status was ascertained through self-reported medical history verification. diabetes mellitus (DM) was operationally defined according to prevailing diagnostic criteria, requiring fulfillment of at least one of the following: fasting plasma glucose ≥ 7.0 mmol/L; HbA1c ≥ 6.5%; 2-hour postprandial glucose ≥ 11.1 mmol/L during oral glucose tolerance testing; or physician-confirmed diagnosis with concurrent antidiabetic pharmacotherapy (insulin or oral hypoglycemic agents). Hyperlipidemia was defined as having a total cholesterol level of ≥6.2 mmol/L (or ≥260 mg/dL), triglycerides ≥ 1.7 mmol/L (or ≥150 mg/dL), and low-density lipoprotein cholesterol (LDL-C) ≥ 4.1 mmol/L (or ≥160 mg/dL). Chronic kidney disease (CKD) was defined as an estimated glomerular filtration rate of <60 mL/min/1.73 m^2^ and/or a urinary albumin-to-creatinine ratio > 30 mg/g.^[27]^

#### 2.1.4. Statistical analysis

All statistical analyses were conducted using mobile examination center sampling weights. Continuous variables were summarized as means ± standard errors and compared using independent samples *t*-tests. Categorical variables were expressed as frequencies with percentages and standard errors, and analyzed through chi-square tests. Logistic regression analysis employed a hierarchical 3-model structure: model 1 (unadjusted) contained no covariates; model 2 adjusted for demographic factors including age, sex, education level, race/ethnicity, and BMI; model 3 incorporated comprehensive adjustments for age, sex, poverty index, education, alcohol consumption status, smoking status, PHQ-9-scores, SII, hemoglobin, creatinine, HbA1c, DM, coronary heart disease, CKD, and congestive heart failure. We conducted a sensitivity analysis to address potential bias arising from missing covariate data by excluding participants with incomplete information and reevaluating the association between FTUs and hypertension

Restricted cubic spline (RCS) regression was employed to flexibly characterize the nonlinear relationship between FTUs and hypertension, using the FTUs level as the reference knot position. A 3-path mediation framework based on model 3 covariates was implemented to assess the potential mediating role of HbA1c in the FTUs- hypertension association. This analytical approach quantified: the total effect (α) of FTUs on hypertension, the exposure-mediator effect (β₁) of FTUs on HbA1c levels, and the mediator-outcome effect (β₂) of HbA1c on hypertension. The direct effect was derived using the counterfactual framework formula: direct effect = α − (β₁ × β₂).^[28]^ Subgroup analyses were performed categorized by age, sex and PHQ-9 score to examine potential effect modification. Statistical significance was defined as 2-tailed *P*-values < .05. All analyses were conducted using R 4.2.2.

## 3. Results

The participants from the 2005–2018 NHANES with baseline characteristics in Table 1. Significant intergroup disparities emerged across multiple domains: age, sex, race, education level, poverty index, BMI, HbA1c, FTUs, diabetes status, hyperlipidemia, alkaline phosphatase, albumin, lymphocyte count, monocyte count, neutrophil count, SII, hemoglobin, creatinine, smoking status, alcohol consumption, PHQ-9-scores, CKD and coronary heart disease.

**Table 1:** Characteristics of the participants.

Characteristics total (N = 25,638)	Healthy-controls	Hypertension patients	*P*-value
Age*	42.062 (0.254)	56.805 (0.269)	<.001
Sex†			.013
Female	7365 (51.165)	5334 (49.179)	
Male	7348 (48.835)	5591 (50.882)	
Smoke†			<.001
Former	3010 (21.533)	3389 (31.575)	
Never	8489 (57.773)	5456 (49.810)	
Current	3214 (20.674)	2080 (18.615)	
Drinking†			<.001
Former	1846 (10.543)	2301 (17.322)	
Heavy	3425 (23.642)	1710 (17.120)	
Mild	5010 (36.785)	3876 (38.939)	
Moderate	2601 (19.401)	1451 (15.621)	
Never	1831 (9.629)	1587 (10.998)	
EDU†			<.001
<High school	2979 (13.104)	2810 (16.463)	
Collage	8556 (65.380)	5421 (58.291)	
High school	3178 (21.606)	2694 (25.246)	
Race†			<.001
Black	2480 (8.589)	2756 (12.316)	
White	6528 (69.200)	5047 (72.189)	
Other	5705 (22.211)	3122 (72.189)	
PHQ-9 score*	2.757 (0.047)	3.308 (0.660)	<.001
BMI (kg m^2^)*	27.848 (0.095)	31.088 (0.103)	<.001
FTUs*			<.001
FTUs < 3	2383 (11.559)	4338 (29.781)	
3 ≤ FTUs ≤ 9	3209 (18.492)	2998 (26.868)	
10 ≤ FTUs ≤ 12	9121 (69.949)	3589 (43.352)	
Poverty index*	3.120 (0.039)	3.049 (0.037)	.024
HbA1c*	5.438 (0.009)	5.880 (0.014)	<.001
ALP*	65.713 (0.279)	71.795 (0.385)	<.001
Albumin (g/L)*	43.021 (0.054)	42.210 (0.062)	<.001
Lymphocyte*	30.835 (0.107)	29.181 (0.154)	<.001
Monocyte*	7.898 (0.031)	8.209 (0.044)	<.001
Neutrophil*	57.825 (0.120)	58.995 (0.160)	<.001
Hemoglobin*	14.323 (0.025)	14.271 (0.031)	.031
SII*	525.335 (3.918)	572.754 (5.28)	<.001
Creatinine*	0.859 (0.003)	0.947 (0.005)	<.001
DM†	1379 (6.784)	3352 (25.586)	<.001
CKD†			<.001
No	13,447 (92.767)	7711 (75.257)	
Yes	1266 (7.233)	2998 (26.868)	
CHD†			<.001
No	14,509(98.766)	10,146(93.441)	
Yes	204 (1.234)	779 (6.559)	
Hyperlipidemia†			<.001
No	5287 (36.474)	1981 (17.214)	
Yes	9426 (63.526)	8944 (82.786)	

*P* < .05 indicates statistical significance.

ALP = alkaline phosphatase, BMI = body mass index, CHD = coronary heart disease, CKD = chronic kidney disease, DM = diabetes mellitus, EDU = education, FTU = functional tooth unit, HbA1c = metabolic markers: glycated hemoglobin, PHQ-9 = 9-item Patient Health Questionnaire, SD = standard deviation, SII = systemic immune-inflammation index.

* Mean ± SD for continuous variables: *P* value was calculated by linear regression model.

† % for categorical variables: *P* value was calculated by chi-square test.

Table 2 presents the multivariable-adjusted logistic regression results examining the association between FTUs and hypertension risk. Each standard deviation increase in FTUs levels was associated with a 1.5% reduction in hypertension risk (adjusted odds ratio [OR] = 0.985, 95% confidence interval [CI]: 0.975–0.996). Participants in the highest FTUs grounp (10 ≤ FTUs ≤ 12) demonstrated significantly lower hypertension risk compared to the lowest grounp (FTUs < 3 units), with an adjusted OR of 0.82 (95% CI: 0.72–0.92, *P* < .001) after full covariate adjustment.

**Table 2:** Association between functional tooth units and hypertension.

Character	Model 1		Model 2		Model 3	
	OR (95% CI)	*P*-value	OR (95% CI)	*P*-value	OR (95% CI)	*P*-value
FTUs	0.872 (0.865, 0.879)	<.001	0.971 (0.961, 0.980)	<.001	0.985 (0.975, 0.996)	.006
FTUs < 3	Ref		Ref		Ref	
3 ≤ FTUs ≤ 9	0.56 (0.51, 0.62)	<.001	0.89 (0.80, 1.00)	.05	1.02 (0.90, 1.16)	.74
10 ≤ FTUs ≤ 12	0.24 (0.22, 0.26)	<.001	0.69 (0.862, 0.77)	<.001	0.82 (0.72, 0.92)	.001

Model 1: no variables were adjusted; model 2: adjusted for age, sex, education, BMI; model 3: adjusted for age, sex, race, poverty index, education, alcohol status, smoking status, PHQ-9-scores, SII, hemoglobin, creatinine, HbA1c, DM, CKD, and congestive heart failure.

BMI = body mass index, CI = confidence interval, CKD = chronic kidney disease, DM = diabetes mellitus, FTU = functional tooth unit, HbA1c = metabolic markers: glycated hemoglobin, OR = odds ratio, PHQ-9 = 9-item Patient Health Questionnaire, Ref = reference, SII = systemic immune-inflammation index.

**Figure 2.**
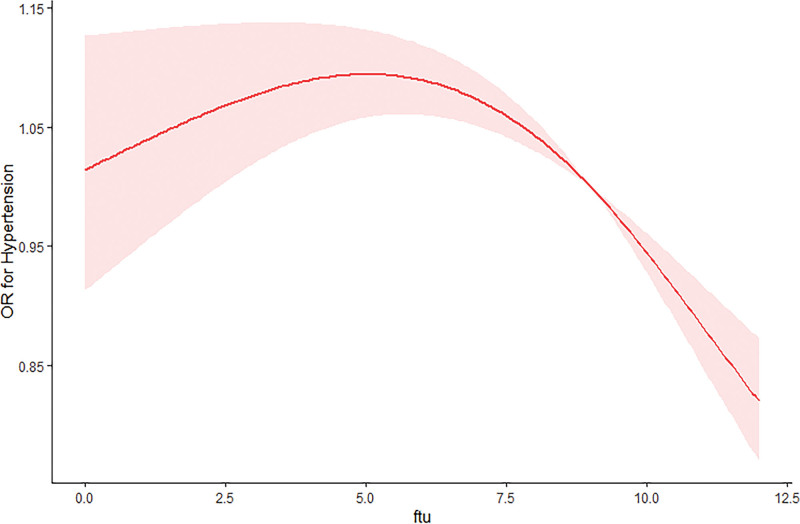
Relationship between FTUs width and hypertension. FTU = functional tooth unit, OR = odds ratio.

The RCS revealed a threshold effect of FTUs on hypertension risk at 5.0 units (Fig. 2). Below this threshold, each unit increase in FTUs showed non-significant association with hypertension (OR = 1.020, 95% CI: 0.979–1.066, *P* = .386). Conversely, above the threshold value, every incremental FTU unit was associated with 3.9% reduced hypertension risk (OR = 0.961, 95% CI: 0.940–0.983, *P* < .001).

**Figure 3.**
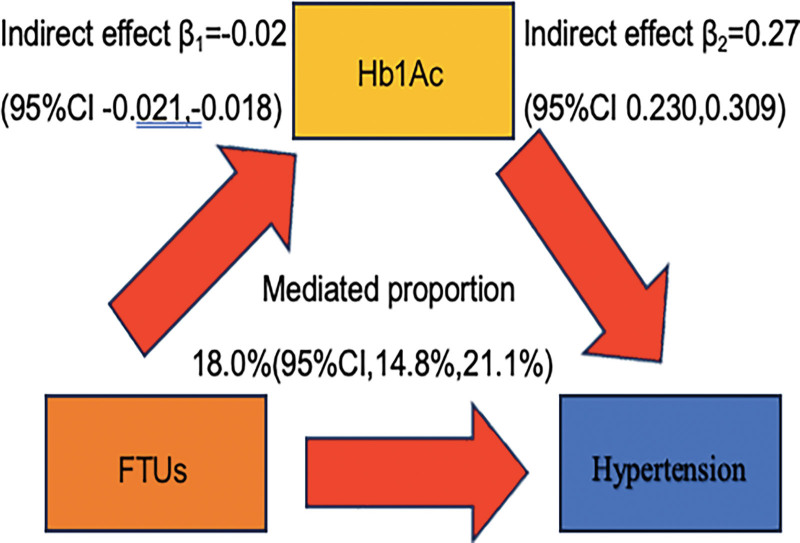
The mediating effect analysis of FTUs and hypertension. CI = confidence interval, FTU = functional tooth unit, HbA1c = metabolic markers: glycated hemoglobin.

The mediation analysis was conducted to assess HbA1c’s mediating role in the masticatory function-hypertension association (Fig. 3). The results indicated statistically significant partial mediation, with HbA1c accounting for 18.0% (95% CI: 14.8–21.1, *P* < .001) of the total effect through indirect pathways.

Subgroup interaction analysis revealed sex-specific and age-stratified differences in the association between FTUs and hypertension risk. However, the interaction *P*-values (.017–.023) were close to the threshold for statistical significance. Thus, these results should be interpreted with caution and regarded as hypothesis-generating rather than confirmatory. The protective effect of FTUs was more pronounced in younger adults (<60 years: adjusted OR = 0.922, 95% CI: 0.909–0.934) compared to older adults (≥60 years: OR = 0.970, 95% CI: 0.951–0.989), sex-stratified models demonstrated stronger associations in women (OR = 0.976, 95% CI: 0.960–0.991) than men (OR = 0.983, 95% CI: 0.969–0.997), respectively (Table 3).

**Table 3:** Subgroup analysis of the association between functional tooth units and hypertension.

Character	OR	95% CI	*P*-value	*P* for interaction
PHQ-9				.26
No	0.997	0.996–0.999	<.001	
Yes	0.992	0.963–1.023	.619	
Aged				<.001
<60	0.922	0.909–0.934	<.001	
>60	0.970	0.951–0.989	.003	
Sex				<.001
Female	0.976	0.960–0.991	.003	
Male	0.983	0.969–0.998	.024	

CI = confidence interval, OR = odds ratio, PHQ-9 = 9-item Patient Health Questionnaire.

As shown in Table 4, we conducted a sensitivity analysis by reassessing the changes in masticatory function and found the associations between FTUs and hypertension remained robust, supporting a persistent association between lower FTUs and an increased risk of hypertension.

**Table 4:** Sensitivity analysis.

Character	Model 1		Model 2		Model 3	
	OR (95% CI)	*P*-value	OR (95% CI)	*P*-value	OR (95% CI)	*P*-value
FTUs	0.861 (0.855–0.867)	<.001	0.963 (0.956–0.971)	<.001	0.982 (0.973–0.990)	<.001
FTUs < 3	Ref		Ref		Ref	
3 ≤ FTUs ≤ 9	0.518 (0.473–0.568)	<.001	0.859 (0.772–0.957)	.006	0.968 (0.865–1.084)	.569
10 ≤ FTUs ≤ 12	0.210 (0.193–0.227)	<.001	0.682 (0.772–0.957)	<.001	0.837 (0.760–0.922)	.001

Model 1: no variables were adjusted; model 2: adjusted for age, sex, race, BMI; model 3: adjusted for age, sex, race, BMI, education, alcohol status, smoking status, SII, hemoglobin, creatinine, ALP, DM, platelet, hyperlipidemia, albumin, CKD, and congestive heart failure.

ALP = alkaline phosphatase, BMI = body mass index, CI = confidence interval, CKD = chronic kidney disease, DM = diabetes mellitus, FTU = functional tooth unit, OR = odds ratio, Ref = reference, SII = systemic immune-inflammation index.

## 4. Discussion

In this comprehensive US-based population study, we identified a significant association between FTUs and hypertension risk, with participants exhibiting fewer FTUs demonstrating increased susceptibility to hypertension development. The dose–response relationship manifested a distinct inverted U-shaped pattern, revealing nonlinear progression characteristics.

Existing research has predominantly focused on the association between tooth loss and hypertensive disorders. In a systematic review and meta-analysis conducted by Tada et al, individuals with significant tooth loss demonstrated elevated hypertension prevalence, accompanied by markedly higher systolic blood pressure measurements compared to those with fewer remaining teeth.^[13]^ A population-based nationwide cohort study conducted in South Korea demonstrated a significant dose-dependent association between tooth loss quantity and hypertension incidence (RR = 2.26; 95% CI: 1.24–4.10, *P *= .007).^[29]^ A population-based cross-sectional study conducted by Da et al among community-dwelling older adults in Shanghai revealed a significant positive association between tooth loss burden and hypertension prevalence in this population.^[15]^ The Shimane CoHRE investigation revealed an inverse correlation between preserved masticatory function and hypertension risk, while demonstrating that edentulism without prosthetic rehabilitation significantly increased hypertension susceptibility. Notably, this study was limited by its reliance on subjective assessments rather than standardized objective metrics such as FTUs for masticatory performance evaluation.^[30]^ This investigation substantiates the regression findings through concordant evidence. Our analysis demonstrated that diminished FTUs were independently associated with elevated hypertension risk following multivariable adjustment. Notably, when compared to individuals with impaired masticatory function (FTUs < 3), those maintaining optimal masticatory capacity (FTUs 10–12) exhibited a 28% reduced risk of hypertension (OR = 0.82, 95% CI: 0.72–0.92). Nonlinear modeling in our study revealed a threshold effect in the association between FTUs and hypertension risk, with segmented regression identifying a distinct inflection point at FTUs = 5. Below this threshold, each unit increase in FTUs showed non-significant association with hypertension (OR = 1.020, 95% CI: 0.979–1.066, *P* = .386). Conversely, above the threshold value, every incremental FTU unit was associated with 3.9% reduced hypertension risk (OR = 0.961, 95% CI: 0.940–0.983, *P* < .001).

A population-based cross-sectional investigation conducted by Oohira et al identified a significant inverse association between FTUs and HbA1c levels suggesting potential metabolic implications of masticatory dysfunction.^[31]^ A cross-sectional study by Fujishiro et al reported that participants with chewing difficulties had significantly higher HbA1c levels compared with those without such difficulties.^[32]^ The aforementioned findings demonstrate a significant correlation between masticatory function and both DM progression and HbA1c elevation. Mechanistically, diabetes and insulin resistance exacerbate hypertension prevalence through multifactorial pathways, including dysregulated activation of the renin-angiotensin-aldosterone system and sympathetic nervous systemenhanced renal sodium reabsorption via endothelial sodium channel activation, mitochondrial dysfunction-induced oxidative stress, chronic inflammatory responses, gut microbiota dysbiosis, aberrant exosomal microRNA profiles, and upregulated renal sodium-glucose cotransporter 2 activity.^[33]^ Our mediational analysis revealed that reduced FTUs mediated hypertension risk through elevated HbA1c levels. This mechanistic pathway suggests that impaired masticatory function may contribute to hypertension pathogenesis via hyperglycemia-induced mechanisms, establishing a potential causal chain from oral health deterioration to metabolic dysregulation.

Beyond its established association with hyperglycemia-related mechanisms, diminished masticatory capacity may independently contribute to hypertension pathogenesis through distinct biological pathways.

Periodontitis, the primary cause of tooth loss, is an inflammatory condition affecting the teeth’s supporting tissues due to bacterial infection,^[34]^ localized periodontal inflammation may trigger systemic inflammatory responses through C-reactive protein and key proinflammatory mediators including TNF-α, IL-1β and IL-6. These mechanisms compromise endothelial-dependent blood pressure homeostasis, potentially exacerbating hypertension pathogenesis.^[35–37]^ Periodontitis-associated pathogens, including Porphyromonas gingivalis, gain access to the systemic circulation through transient bacteremia. These microorganisms subsequently invade vascular endothelial walls, triggering inflammatory responses that ultimately contribute to atherosclerotic plaque formation.^[38]^ Impaired masticatory function results in preferential consumption of energy- dense foods while reducing the intake of vitamin- and fiber-rich fruits and vegetables, this dietary shift creates nutrient deficiencies and dietary imbalances that contribute mechanistically to the development of chronic hypertension.^[39]^ Diminished dietary consumption decreases meal-associated thermogenesis and suppresses histaminergic neuronal activity. These physiological alterations promote adipogenesis through metabolic dysregulation, consequently establishing a pathogenic pathway that elevates risk for obesity-associated hypertension.^[13]^ However, emerging evidence indicates that chronic systemic diseases also significantly influence periodontiti.^[40]^ Numerous studies have established DM not only as an independent risk factor for periodontitis prevalence, but also as a critical modifier that exacerbates disease progression.^[41,42]^ Samanovic et al demonstrated a significant positive correlation between hypertension and increased radiographic area in periodontitis through their analysis,^[43]^ In addition, multiple study revealed that cardiovascular disease patients exhibited a 3-fold elevated risk of developing periodontitis compared to non-cardiovascular disease controls.^[44]^ Subgroup interaction analysis revealed significant sex-specific and age-stratified variations in the association between FTUs and hypertension risk. This finding aligns with epidemiological evidence demonstrating sex-based disparities in hypertension prevalence across populations. The Heart Disease and Stroke Statistics 2021 update reported an age-adjusted hypertension prevalence of 51.7% among males and 42.8% among females in US adults aged ≥ 20 years during the 2015 to 2018 surveillance period. This sex-specific disparity is hypothesized to originate from endocrine-mediated pathophysiological mechanisms.^[45]^ Research has demonstrated that estrogen regulates blood pressure through multiple biological mechanisms, including sympathetic nervous systemenhanced modulation, renin-angiotensin-aldosterone system regulation, oxidative stress mitigation, inflammatory response control, and endothelial function maintenance. These pathways collectively exert protective effects on blood pressure homeostasis in premenopausal women.^[46,47]^ Age-related elevation in blood pressure is primarily mediated by 2 pathophysiological mechanisms: progressive decline in vascular compliance and age-associated hemodynamic alterations.^[48]^ These factors serve as modulators of hypertension risk and mediate intergroup disparities in the FTUs-hypertension association. This study demonstrates notable methodological advancements compared to prior investigations. Firstly, the research enhances statistical power through the utilization of a nationally representative, large-scale sample. Furthermore, to ensure analytical robustness, multiple analytical approaches were employed including multivariate logistic regression, RCS analysis and subgroup analyses. These methodologies collectively establish a significant association between masticatory function and hypertension risk, providing critical insights for public health strategies targeting hypertension prevention and clinical management. However, this study has several noteworthy limitations that should be acknowledged. First, the calculation of FTUs relies on occlusal contact pattern assumptions without accounting for dental mobility, due to insufficient clinical data. This methodological constraint may result in imprecise characterization of masticatory function. Second, all epidemiological data were acquired through NHANES protocols involving self-reported data and mobile screening, which may introduce measurement inaccuracies and compromise the objectivity of findings. Most significantly, the cross-sectional design inherently precludes causal inference between masticatory function and hypertension incidence.

Therefore, prospective studies are needed to confirm the association between masticatory function and hypertension.

## 5. Conclusion

Our findings suggest that higher FTUs may lower the risk of hypertension via the HbA1c pathway.

## Author contributions

**Conceptualization:** Hao Guo, Tian Lv.

**Data curation:** Hao Guo, Hang Yang, Jie Li.

**Project administration:** Hao Guo.

**Writing** – **original draft:** Hao Guo.

**Writing** – **review & editing:** Tian Lv.
